# Carbon Nanotubes in historical and future perspective Summary of an Extended Session at Carbon 2008 in Nagano (JP)

**DOI:** 10.1186/1743-8977-5-21

**Published:** 2008-12-10

**Authors:** Morinobu Endo, Shuji Tsuruoka, Gaku Ichihara

**Affiliations:** 1 Electrical and Electronic Engineering, Faculty of Engineering, Shinshu University, Nagano, Japan; 2 Functional Materials Division, Mitsui & Co., Ltd., Tokyo, Japan; 3 Occupational and Environmental Health, Nagoya University Graduate School of Medicine, Nagoya, Japan

## Abstract

The extended session on Biological Evaluations with Carbon Nanotubes was held on 18 July, 2008 in Nagano as a part of the International Carbon 2008 Conference. During this session researchers and regulators discussed recent publications that have shown significant hazards of carbon nanotubes in animal models and have received wide coverage in the lay press. The discussion focused on significance and interpretation of the data, their meaning to further development, and prevention of exposure at the workplace. The paper of Poland et al was presented and detailed by Dr. Duffin, a senior researcher at the ELEGI-COLT lab at Edinburgh University (UK). Dr. Takagi and his team did not share our discussion although they were invited to do so.

## Background

Carbon is an old material originally used as a mineral and only recently available as an engineered material. Carbon materials have been utilized in various fields including daily life applications such as pencils, nuclear plants, tires and batteries. During the last decades, major breakthroughs in carbon science and technology have delivered new carbon materials such as carbon fibers, fullerenes and carbon nanotubes, and some envisage the carbon age to replace the silica era. Updates in carbon science developments have always been communicated in the past at the American Carbon Society, formerly The American Carbon Committee. This society was established with the purpose of organizing conferences and workshops in order to promote interdisciplinary research and technology in the field of carbon science. The Carbon Conferences are the major international conferences encompassing all areas of carbon science and technologies. The Carbon Conference was, for the first time, held in 1953 and it has continued biennially. In recent years, carbon nanotubes have provoked considerable interest among toxicologists and it was therefore the purpose of the most recent edition of the carbon meeting in Nagano to bring together researcher from the field of carbon science and toxicology in a special, extra session to its program. As chairs and organizers of this program we would like to communicate the goal and outcomes of this session to a broader public.

## Report

Earlier studies with single-wall carbon nanotubes have been done with CNT instilled into the lung of rodents. These studies showed that SWCNT cause similar classical lesions in the lung as crystalline silica, including granuloma, inflammation and interstitial fibrosis [1-5]. Only in the first half of this year, two papers were published that focused on the toxicity which can typically be caused by materials with fibrous shape. Both studies investigated the acute [6] and chronic [7] response of the peritoneal mesothelium of mice after installation of different types of nanotubes and reference materials. In addition a risk evaluation was published by the National Institute for Occupational Safety and Health in US urging the use of crystalline silica TLV for single-wall nanotubes. These studies and reports have stirred both the carbon society as well those working in nanotechnology in general.

Above studies and their findings were the major topics of the extended session on Biological Evaluations with Carbon Nanotubes held on 18 July, 2008 in Nagano as a part of the International Carbon 2008 Conference. During this session researchers and regulators discussed recent publications that have shown significant hazards of carbon nanotubes in animal models and have received wide coverage in the lay press. The discussion focused on significance and interpretation of the data, their meaning to further development, and prevention of exposure at the workplace. The paper of Poland et al was presented and detailed by Dr. Duffin, a senior researcher at the ELEGI-COLT lab at Edinburgh University (UK). Dr. Takagi and his team did not share our discussion although they were invited to do so.

The main objectives of the workshop were to give direction for and facilitate future research programs and studies on carbon nanotubes. More specifically these include:A. Establishing protocols for evaluation of biological effects of CNT

1. To clarify and review carefully the natural exposure scenario to CNT. Efforts should be made to mimic as well possible workplace exposure doses and particle characteristics2. To integrate toxicology and material science to pursue biological evaluations with nano materials such as carbon nanotubes. It does not make sense to use agglomerated fibers preparations, while a mineral fiber paradigm is investigated.

3. To harmonize and standardize techniques including particle dispersion methods, positive and/or negative references particles, animal models and pathological evaluation techniques.

B. To establish a databank of benchmark samples of relevant CNT1. The databank should contain several relevant samples of MWCNT and SWCNT in addition to Mitsui MWNT-7 used by toxicology studies so far.2. To allow evaluation of commercially relevant products rather than small production samples synthesized in the laboratory.

3. It seems a good recommendation to use micro size silica as a reference due to the large toxicological database for this particle.

C. To establish and maintain a continuous scientific discussion

1. To have interactive meeting among toxicologists, pathologists and material scientists rather than formal ones.

2. To discuss a way to develop nanocarbon materials for market places, where toxicology provides material science and engineering sustainable carbon materials in future.

The workshop was opened with a presentation of Dr. Rodger Duffin (Univ. of Edinburgh UK) entitled, "The Variable Hazard of Carbon Nanotubes: Length Development Pathogenic Behavior". Dr. Duffin extended the findings as published by his team in Poland et al (2008). Dr. Duffin showed that also lower doses than the initial 50 μg induced short-term inflammation in the mouse peritoneum after injection of MWCNT longer than 15 microns. A dose relationship was established between the amount of neutrophils and doses of 0.1, 0.25, 0.5, 0.75, 1, 5, 10 25 and 50 μg of MWCNT. In addition a further follow-up of mice in the study showed that 6 weeks after injections of MWCNT the size and number of granulomas did increase further and developed into lesions with massive fibrosis present. Interestingly the study was stopped after 6 weeks because animals showed adhesion of peritoneum to endogenous organs and animals were sacrificed for ethical reasons. The group in Edinburgh clearly states that their data confirm the fibre paradigm, but do no yet prove that CNT can cause mesothelioma. The route of administration and the dose are highly artificial and further studies are needed to repeat this by inhalation and realistic exposure scenarios.

Next, Prof. Håkan Wallin from the NRCWE in Denmark gave a presentation entitled, "Research on Carbon Materials at NRCWE for Working Environment". Prof. Wallin explained the importance of new metrics, such as surface area in the expression of toxicity of nanomaterials. It is also necessary to distinguish whether particles reach the deep part of the lung or not. NRCWE is studying the dustiness of nanomaterials and is evaluating exposures at work processes such as CNT handling, production and wear of CNT composites.

Prof. Paul. Borm, from Zuyd University (The Netherlands) gave a presentation entitled, "Carbon Nanomaterials: Opportunities & Health Hazards from a European Perspective". In this presentation he presented recent outcomes of surveys on best-practices and handling of nanomaterials in Dutch and European industry. In addition, he made a thorough comparison of the studies with MWCNT published by Takagi et al [6] and Poland et al [7]. The Poland paper was set-up with a clear hypothesis to confirm or reject the fibre paradigm and designed as a short-term study; it is therefore impossible to draw conclusions from this study on pathological events in a chronic time frame. The dose used (50 μg/mouse) is considerably lower, and better suspended than in the Takagi-paper which used 3 mg/mouse and showed clumps of aggregated MWCNT. Nevertheless both studies have instilled fibre numbers that in previous studies with the same model in rats were positive also for non-carcinogenic fibers such as man-made mineral fibres. The data provided by Dr. Duffin earlier on, do show that also at lower fibre numbers MWCNT elicited a similar response, although quantitatively less. Finally, the Takagi paper is poorly documented with regard to pathological classification of individual animals, and a clear need for independent review of histological sections is needed.

Dr. Junko Nakanishi from the AIST presented preliminary data emerging from Japan's national program on toxicological research of nanomaterials in her lecture entitled, "Our Research Strategy on Nanomaterials EHS for Risk Assessment". In this programme 3 nanomaterials are being tested, including nickel oxide, fullerenes and CNT by chronic inhalation. The data of NiO_2_ and C60 are now emerging and show little translocation to the brain. Interestingly a NOAEL for NiO_2_ of 0.12 mg/m^3^ was found after inhalation, while a NOAEL of 0.67 mg/kg bw (rat) was found after intratracheal instillation of fullerene. No lipid peroxidation was noted in mouse brain after 12 months of C60 inhalation as opposed to earlier studies by Eva Oberdorster in sea-brass [8]. Also other biomarkers for oxidative stress in the brain (Heme oxygenase-1, GSH, Malondialdehyde) were negative. In the next 2 years outcomes for fullerenes and CNT are to be expected

Dr. Sivaram Arepalli from NASA-JSC presented data from NASA's collaborative study with NIOSH entitled, "Comparison of Pulmonary Responses to Single-Walled vs Multi-Walled Carbon Nanotubes" on behalf of V. Castranova (NIOSH). NIOSH results of exposure-response to SWCNT and MWCNT (Mitsui MWNT-7) in pulmonary tests were presented. Both CNTs showed acute inflammation which declined 7 days after exposure. Neurotoxicity appeared in selected parts of the mouse brain after pulmonary exposure but was short lived. All CNTs caused some cardiovascular effects after pulmonary exposure. NASA assumes that CNT hazard is the same as silica, so that similar regulation is suggested for CNTs.

During the panel discussion, Prof. Shozo Koyama showed that pathological evaluation study by subcutaneous implantation based on systemic cytokine analysis as one of the analytical process of CNT toxicological studies from the view points of immunological studies. The discussion further concentrated on future perspectives in development, handling and commercialization of CNT. The experts agreed that current findings certainly present a warning signal to those exploring the use of long MWCNT into customer products. However, further studies are needed to prove that also inhalation of MWCNT can lead to a similar effect in the pleural mesothelium. For that a stepwise approach is recommended taking into account the issues mentioned in this commentary, such as including representative, commercial samples.

In addition, parallel to the development of mineral fibres in the 80-ies and the 90-ies of the last century, the findings provide opportunities to engineer out potential toxicity. Decreasing biodurability, limiting length of building block MWCNT, absolute immobilization in matrix are obvious approaches for the near future. It is anticipated that the contacts made between carbon scientists and toxicologist during the Nagano meeting, will lead to the interdisciplinary interface which is needed for a creative solution towards durable applications of carbon nanotubes.

## Competing interests

The authors declare that they have no competing interests.

## Authors' contributions

EM was the Carbon 2008 Chairperson. ST was as an organizer for the special session. GI was a session chair for the special session.
